# FMS-Like Tyrosine Kinase 3 Ligand Treatment Does Not Ameliorate Experimental Rapidly Progressive Glomerulonephritis

**DOI:** 10.1371/journal.pone.0123118

**Published:** 2015-04-07

**Authors:** Joanna R. Ghali, Kim M. O’Sullivan, Peter J. Eggenhuizen, Stephen R. Holdsworth, A. Richard Kitching

**Affiliations:** 1 Centre for Inflammatory Diseases, Department of Medicine, Monash University, Clayton, Victoria, Australia; 2 Department of Nephrology, Monash Health, Clayton, Victoria, Australia; 3 Department of Paediatric Nephrology, Monash Health, Clayton, Victoria, Australia; International Research and Development Center for Mucosal Vaccine, Institute of Medical Science, The University of Tokyo, JAPAN

## Abstract

Fms-like tyrosine kinase 3-ligand (FL) is a growth factor that may expand dendritic cell and regulatory T cell populations. We hypothesised that FL-induced regulatory T cells would protect mice from experimental rapidly progressive glomerulonephritis. To determine if FL was able to enhance regulatory T cell populations, C57BL/6 mice received 10 days of daily intraperitoneal injections of either FL or phosphate buffered saline. To induce accelerated autologous-phase anti-mouse glomerular basement membrane glomerulonephritis, mice were sensitized to sheep globulin 4 days prior to the induction of glomerulonephritis with sheep anti-mouse glomerular basement membrane globulin, and experiments ended 10 days later. FL was administered before, throughout and during the sensitization phase of this glomerulonephritis model. Renal disease and systemic immunity to the nephritogenic antigen were assessed. FL increased regulatory T cell and plasmacytoid dendritic cell proportions within spleen and lymph nodes. FL administration prior to glomerulonephritis did not protect mice from renal injury. When FL was given throughout the model, FL treated mice had reduced survival, with more interstitial neutrophils and glomerular CD11c+ cells than controls. Systemic immune responses showed increased IL-17A production from splenocytes, with more CD11c+ cells, but reduced plasmacytoid dendritic cell proportions in spleen and lymph nodes, despite increased regulatory T cell proportions. Under homeostatic conditions, FL expanded regulatory T cell and plasmacytoid dendritic cell populations, but FL enhanced systemic inflammatory responses and conventional dendritic cell populations when given during experimental glomerulonephritis, suggesting selective attempts to suppress pathogenic immunity by dendritic cell manipulation may be harmful.

## Introduction

Dendritic cells (DCs) are a heterogeneous population of professional antigen presenting cells, derived initially from common myeloid and common lymphoid progenitors in the bone marrow, which terminally differentiate in lymphoid and non-lymphoid tissues. They are broadly characterized into two groups: conventional or classical DCs (cDCs) and plasmacytoid DCs (pDCs) [1, 2]. In the mouse, cDCs include migratory and lymphoid-tissue resident DCs, which express CD11c and MHC II and are further characterized by the expression of other surface markers, including CD11b, CD103 and CD8. pDCs are found in lymph nodes (LN) or circulating within blood, and are CD11c^low-intermediate^, have low MHC II expression and express other surface markers including B220 and murine pDC antigen 1 (PDCA-1) [3–5]. pDCs promote the maturation of cDCs, regulate antigen specific CD4+ T cell proliferation, effector T cell (Teff) production of IFNγ and CD4+foxp3+ Treg homeostasis in mucosal lymphoid tissue [6]. Fms-like tyrosine kinase 3 (FLT3) ligand (FL) is a growth factor that differentiates, matures and expands DCs following ligation of the FLT3 receptor expressed on the surface of hematopoietic precursors [7, 8]. It is expressed in steady state conditions to maintain cDC and pDCs, produced by hematopoietic and stromal cells [9]. Mice deficient in FL lack DCs [10].

Exogenous administration of FL to mice expands DCs within the spleen and lymphoid tissues, and increases tissue resident DC populations, including those in the thymus and kidney [11–13], as well as expanding regulatory T cell (Tregs) populations [14, 15]. Administration of FL to humans also expands immature myeloid DCs, pDCs and Tregs in peripheral blood, and is being explored as immunotherapy for certain malignancies [16–18].

Rapidly progressive glomerulonephritis (RPGN) is characterized by cellular crescents and fibrinoid necrosis of the glomerular tuft. Current therapies are largely non-specific immunosuppressive agents. There is increasing evidence from animal and human studies that Tregs are protective in RPGN [19–24]. Therefore, we hypothesized that exogenous FL may protect mice in experimental RPGN by suppressing nephritogenic immunity via expansion of pDCs and Tregs.

## Materials and Methods

### Experimental design

Male C57BL/6 mice, aged 6–10 weeks were purchased from Monash Animal Research Platform (Monash University) and were housed in specific pathogen free conditions (Monash Medical Centre Animal Facility, Clayton, Victoria, Australia). Studies were performed in accordance with the National Health and Medical Research Council’s Australian code for the care and use of animals for scientific purposes and were approved by the Monash University Animal Ethics Committee B (Ethics Number MMCB12/42). Aged matched mice were randomly assigned to experimental groups, had free access to water and food throughout experiments and were reviewed daily by both the researchers and animal facility staff. Mice were humanely euthanized with carbon dioxide at the completion of experiments or if mice showed any signs of the following: lethargy, persistent recumbency, hunched posture, rough coat or loss of body condition.

FL–Ig (human/human, BioXcell, West Lebanon, NH, USA; as cited in [25, 26]) was delivered as 10μg in 200μL PBS daily intraperitoneal injections for 10 days, as previously published [15]. Control mice received the same volume of PBS at the same times. Initial assessment of the effect of FL vs PBS on naïve mice was made with 4 mice per group. The model of RPGN used was an accelerated autologous phase anti-glomerular basement membrane (GBM) model (n = 6–9 each group; numbers were determined based on prior experience with this model [19]). Mice were sensitized s.c. with 0.5mg normal sheep globulin (in Freund’s complete adjuvant [FCA]) to the right and left tailbase. Four days later, sheep anti-mouse GBM globulin was injected intravenously into the tail-vein and mice were humanely killed after a further 10 days. For delayed type hypersensitivity (DTH), 0.5mg sheep globulin or horse globulin (as a control) was injected into the right and left hind footpads, respectively, and footpad swelling was measured 24hrs later with a micrometer (n = 4 each group). Statistical analysis was performed on Graphpad Prism 6 software. Data are presented as mean (±SEM) or median (range); assessment of 2 groups was performed with a student’s t test or Mann Whitney test for parametric and non-parametric data, respectively. Differences in survival were assessed with a log-rank test. Significant values were defined as P<0.05.

### Assessment of functional and histological injury

Mice were placed on metabolic cages to collect urine prior to the end of experiments. Proteinuria was assessed by Bradford’s assay. Serum was collected from mice and urea levels were measured. To assess renal histology, 3μm-thick formalin-fixed, tissue processed, then paraffin embedded kidney sections were stained with Periodic Acid Schiff’s reagent. Forty glomeruli were assessed for glomerular segmental necrosis and crescent formation (primary outcome). CD4+ T cells, macrophages, and neutrophils were detected by immunoperoxidase staining of 6μm-thick, periodate lysine paraformaldehyde-fixed, frozen kidney sections as previously described [27]. Primary antibodies used were: CD4+ T cells (anti-CD4, GK1.5), macrophages (anti-CD68, FA/11), neutrophils (anti-Gr-1, RB6-8C5) with isotype controls being IgG2b, IgG2a and IgG2b, respectively. For leukocyte infiltration, 20 glomeruli and 10 interstitial high-powered fields were assessed.

### Assessment of immunity by flow cytometry

Lymph nodes (cervical, axillary, brachial, inguinal, mesenteric and para-aortic, pooled for each animal) and spleens were harvested and single cell suspensions were created. One million cells were stained for flow cytometric analyses using the following antibodies: PDCA-1 (eBioscience, San Diego, CA, USA; Bio927), CD11c (BD Biosciences, North Ryde, NSW, Australia; HL3), CD4 (BD Biosciences; GK1.5), foxp3 (eBioscience; FJK-16s), CD25 (BD Biosciences; PC61). For intracellular cytokine staining, cells were fixed with eBioscience Fixation/Permeabilization concentrate, diluent and staining buffer according to the manufacturer’s protocol. Flow cytometry was performed on BD FACS Canto II and data analysed using FlowJo software (TreeStar, OR, USA).

### Assessment of systemic immune responses to sheep globulin

Cultured cell supernatant was collected after stimulating 4 x 10^6^ splenocytes with 100μg sheep globulin in 1mL RPMI-Complete (containing RPMI, 10% foetal calf serum, 50μM 2ME, 10mL penicillin-streptomycin and 5mL of L-glutamine) for 72 hours. IL-17A, IFNγ and IL-4 concentrations were measured by ELISA [28, 29] and other cytokines were measured with the Mouse Inflammation cytometric bead array (BD Biosciences). EliSpot assays for IFNγ (BD Biosciences) and IL-17A (eBioscience) were performed as previously described [30]. Spots were enumerated using an AID EliSpot platereader and software (v4.0, Autoimmun Diagnostika GmbH, Strassberg, Germany). Mouse anti-sheep IgG antibody levels were measured on diluted serum samples by ELISA [31].

### Assessment of CD11c+ DCs in the kidney

To assess renal DCs, immunofluorescent staining of frozen kidney sections was performed using an anti-CD11c antibody (BD Biosciences; HL3) and mounted with DAPI mounting media (Molecular Probes). Images were acquired on a Nikon C1 confocal laser scan head attached to a Nikon Ti-E inverted microscope (Nikon, Tokyo, Japan) using 488 and 561nm lasers. Renal DCs were assessed in three areas: within glomeruli, in the periglomerular region (within 3 cells of Bowman’s capsule) or within a high-powered interstitial field. Twenty glomeruli and periglomerular regions and 10 high-powered fields were assessed for each animal, with CD11c+ immunofluorescence for each of these regions being analysed by Image J software (NIH, Bethesda, MD, USA).

## Results

### Effects of FL on Tregs and DCs

**Fig 1:**
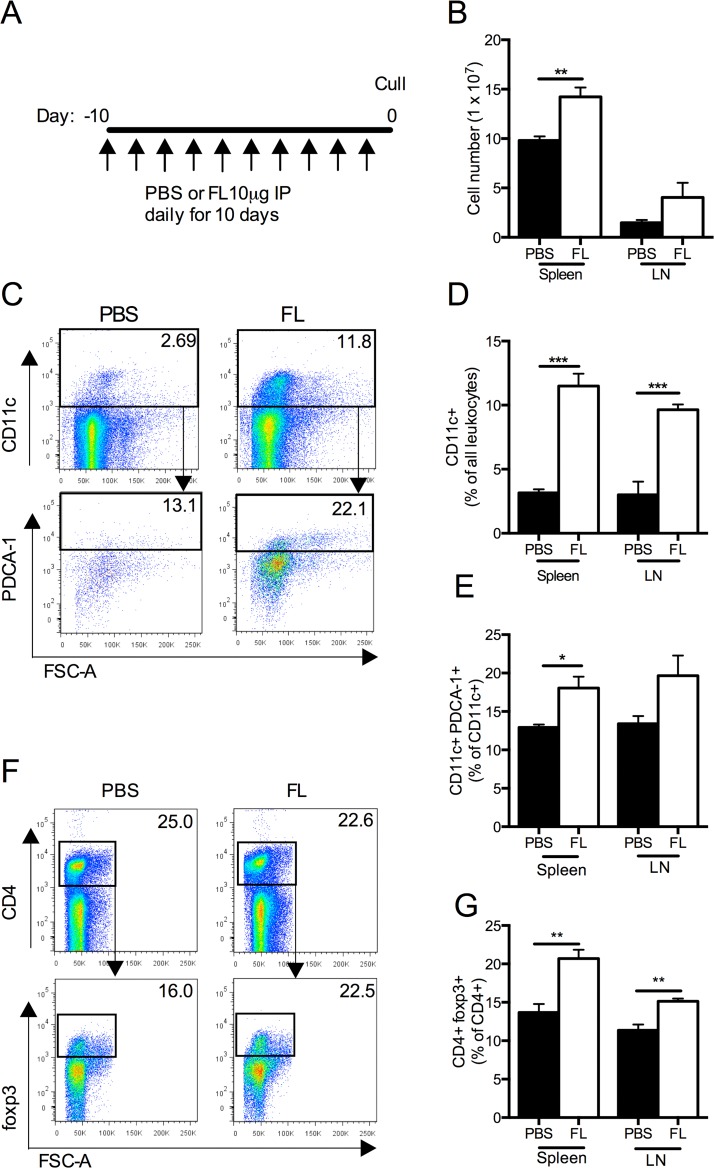
The effects of 10 days of intraperitoneal FL on DC and Treg populations. (A) Experimental design, where FL or PBS was administered intraperitoneally to mice for daily for 10 days. (B) Total spleen and pooled LN cell number. (C) Representative FACS plots showing proportions of pDCs (CD11c+PDCA-1+ cells) in the spleen, gating on all CD11c+ leukocytes. (D) Proportions of CD11c+ cells in the spleen and LN. (E) Proportions of pDCs in the spleen and LN. (F) Representative FACS plots showing Tregs in the spleen, gating on lymphocytes, staining for CD4+ and foxp3+ cells. (G) Proportion of Tregs in the spleen and LN. Black bars represent PBS treated mice. White bars represent FL treated mice. n = 4 per group. *P<0.05, **P<0.01, ***P<0.001.

Daily administration of FL for 10 days (Fig 1A) macroscopically increased spleen size and the size of all LN groups. When quantified, there were increased splenic cell numbers, with a trend towards increased cell numbers in pooled LN (Fig 1B). CD11c+ cells were also increased in the spleen and LN after FL administration (Fig 1C and 1D). Proportions of pDCs (CD11c+PDCA-1+/CD11c+ cells) were significantly elevated in the spleen, with a trend towards increase in the LN (Fig 1C and 1E). The proportion of Tregs (CD4+foxp3+/CD4+ cells) in FL treated animals was significantly increased (Fig 1F and 1G).

**Fig 2:**
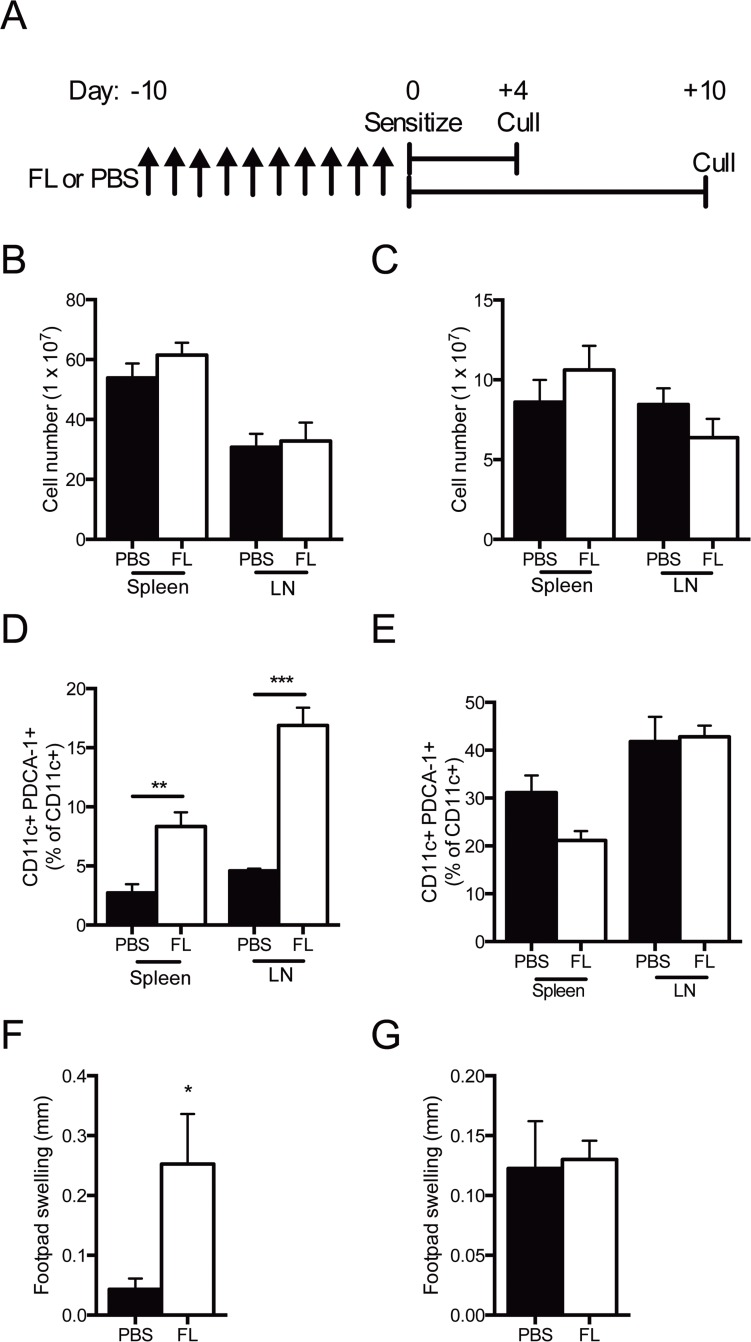
FL therapy expands pDCs but increases delayed type hypersensitivity four days after sensitization to sheep globulin. (A) Experimental design. (B, C) Total spleen and LN cell number 4 and 10 days after sensitization, respectively. (D, E) Proportion of pDCs in the spleen and LN 4 and 10 days post-sensitization to sheep globulin respectively. (F, G) Footpad swelling in mice 4 and 10 days post-sensitization to sheep globulin, respectively. Black bars represent PBS treated mice. White bars represent FL treated mice. n = 4 per group. *P<0.05, **P<0.01.

To determine if increased populations of pDCs and Tregs induced by FL protected mice from generating effector T cell responses, PBS or FL was administered to mice for 10 days, then mice were sensitized to sheep globulin. Mice were then culled 4 or 10 days after sensitization (Fig 2A). At 4 and 10 days post-sensitization, the numbers of splenocytes and LN cells were not different between PBS and FL treated mice (Fig 2B and 2C). Four days after sensitization to sheep globulin, the proportion of CD11c+ cells remained elevated in the LN, but not the spleen of FL treated mice (spleen PBS 20.9±2.5 vs FL 19.4±2.6%, P = 0.7; LN PBS 6.6±0.3 vs FL 9.6±0.4%, P<0.005). FL treated mice had higher proportions of pDCs in spleen and LN (Fig 2D), Ten days after sensitization to sheep globulin, FL treated mice had a reduced proportion of CD11c+ cells in the LN (spleen PBS 4.3±2.2 vs FL 2.4±0.1%, P = 0.44; LN PBS 1.2±0.03 vs FL 0.9±0.02%, P<0.001), but there was no longer any detectable difference in pDC proportions (Fig 2E). No significant differences in Treg proportions were identified four days after sheep globulin sensitization between mice that had been treated with PBS or FL (spleen PBS 7.2±0.8 vs FL 8.6±0.3%, P = 0.16; LN PBS 8.6±0.1 vs FL 9.5±0.5%, P = 0.11). Four days after sheep globulin sensitisation, FL mice developed increased dermal DTH to sheep globulin, suggesting elevated pDC populations did not suppress antigen-specific immunity, but rather enhanced effector T cell function (Fig 2F). When mice were challenged with sheep globulin 10 days after sensitization, DTH was present to a similar degree in PBS and FL treated groups (Fig 2G).

### FL administered prior to the induction of nephritogenic immunity

**Fig 3:**
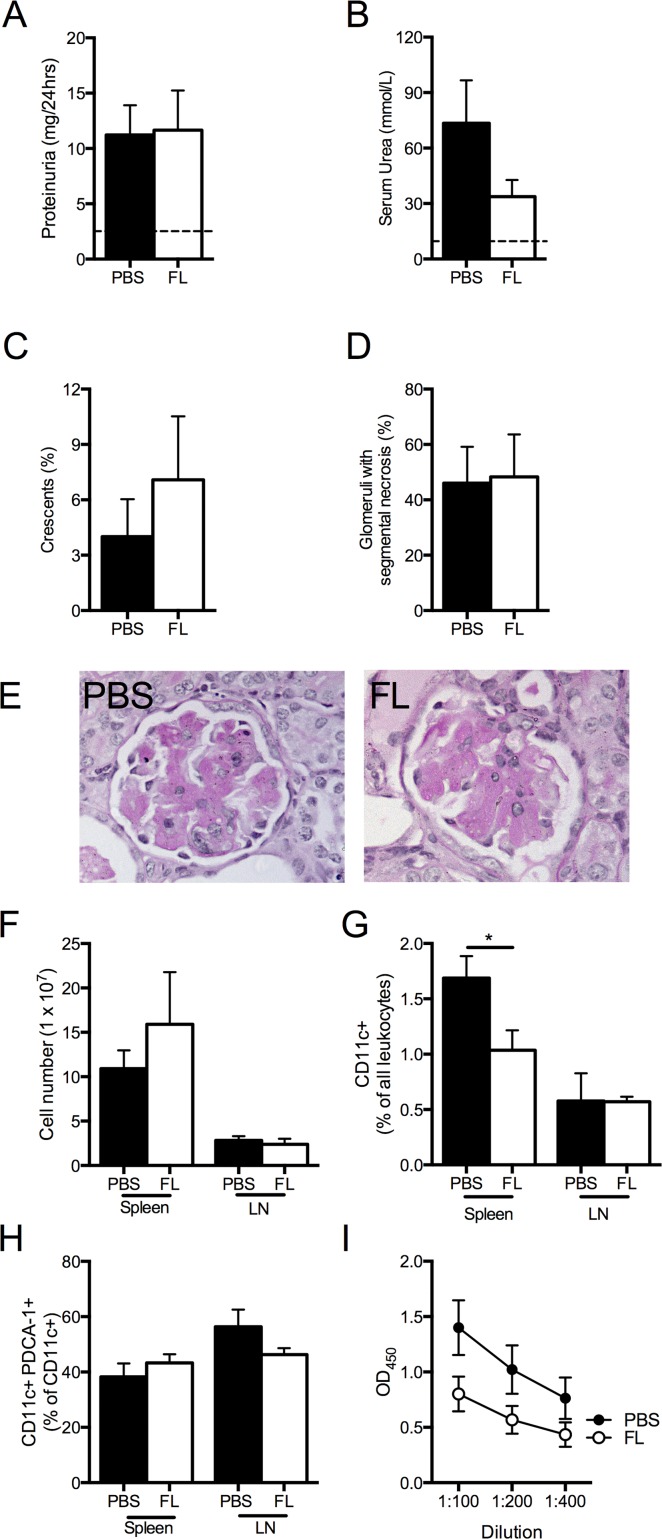
FL administered before accelerated autologous phase anti-GBM disease did not protect against nephritis, but altered systemic immunity. (A) Proteinuria (dotted line represents measured level in non-nephritic WT mice, n = 4). (B) Serum urea (dotted line represents measured level in non-nephritic WT mice, n = 4). (C) Proportions of glomeruli with crescents and (D) glomerular segmental necrosis. (E) Representative images of glomeruli from PBS and FL treated mice taken at high power (400x, PAS stain). (F) Total spleen and LN cell number. (G) Proportion of CD11c+ cells from leukocytes in spleen and LN. (H) Proportion of pDCs in spleen and LN. (I) Serum mouse anti-sheep IgG antibody levels. Black bars represent PBS treated mice. White bars represent FL treated mice. OD, optical density. n = 6 per group. *P<0.05.

When FL or PBS was administered for 10 days before the model of RPGN (with treatment ending the day before mice were immunized with sheep globulin), injury was similar in both groups of mice. No significant differences in renal functional injury (proteinuria or urea; Fig 3A and 3B) or histological damage (glomerular crescents or segmental necrosis; Fig 3C–3E) were found. While the total number of splenocytes or LN cells was not different between groups, the proportion of splenic CD11c+ cells was reduced in FL treated mice (Fig 3F and 3G), but unaltered in the LN between groups. There was no difference in the proportion of pDCs (Fig 3H), nor was there a difference in the proportion of activated T cells (CD4+CD25+/CD4+ cells) in the spleen and LN between the two groups (spleen PBS 4.4±0.4 vs FL 5.5±0.4%, P = 0.12; LN PBS 34.1±3.6 vs 35.9±2.8%, P = 0.7). With respect to cellular immune responses, FL treated mice had reduced IL-17A levels in sheep globulin stimulated splenocyte supernatant compared to controls, but no differences were found in IFNγ, IL-4 or IL-10 concentrations (Table 1); TNF, IL-12p70 and IL-6 concentrations were below the threshold for detection in both groups. FL treated animals had a trend towards lower serum mouse anti-sheep immunoglobulin titres (Fig 3I). Therefore, although FL did not protect mice when given prior to the induction of crescentic GN, some elements of cellular and humoral immune responses were attenuated by FL therapy, suggesting that FL might alter renal injury if delivered throughout the entire course of this disease model.

**Table 1:** Cytokine concentrations in stimulated splenocyte supernatant for mice receiving PBS or FL for 10 days prior to commencement of the anti-GBM disease model.

	Concentration (pg/mL)		
Cytokine	PBS (n = 5)	FL (n = 7)	P
IL-17A	768 ± 130	290 ± 32	0.0019
IFNγ	232 ± 53	332 ± 211	0.63
IL-4	1381 ± 522	710 ± 231	0.22
IL-10	0 (0–12)	0 (0–293)	0.92

Data presented as mean±SEM, except for IL-10, presented as median (range) as data is non-parametric. TNF, IL-12p70 and IL-6 were not detectable.

### FL treatment beginning at sensitization, continuing throughout the disease model

**Fig 4:**
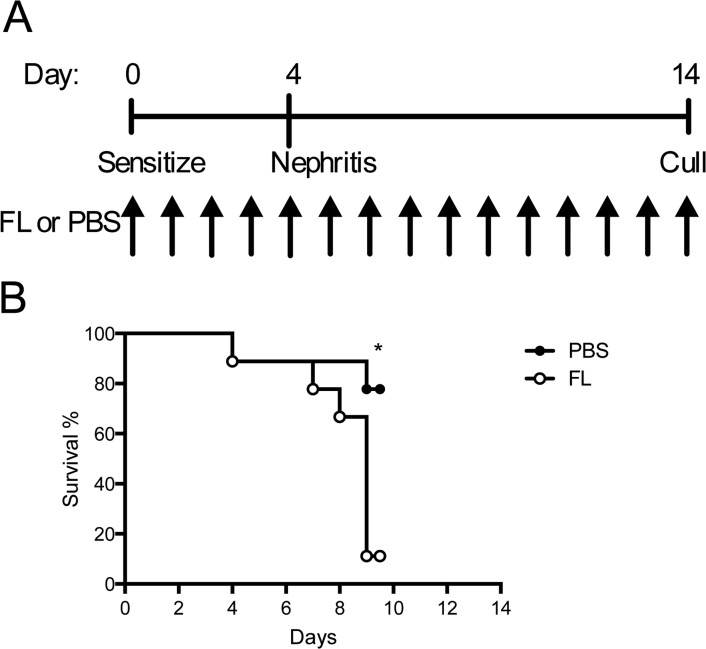
FL administered throughout accelerated autologous phase anti-GBM disease reduced mouse survival. (A) Experimental design. (B) Survival following the induction of nephritis. Black bars represent PBS treated mice. White bars represent FL treated mice. n = 9 per group. *P<0.05.

**Fig 5:**
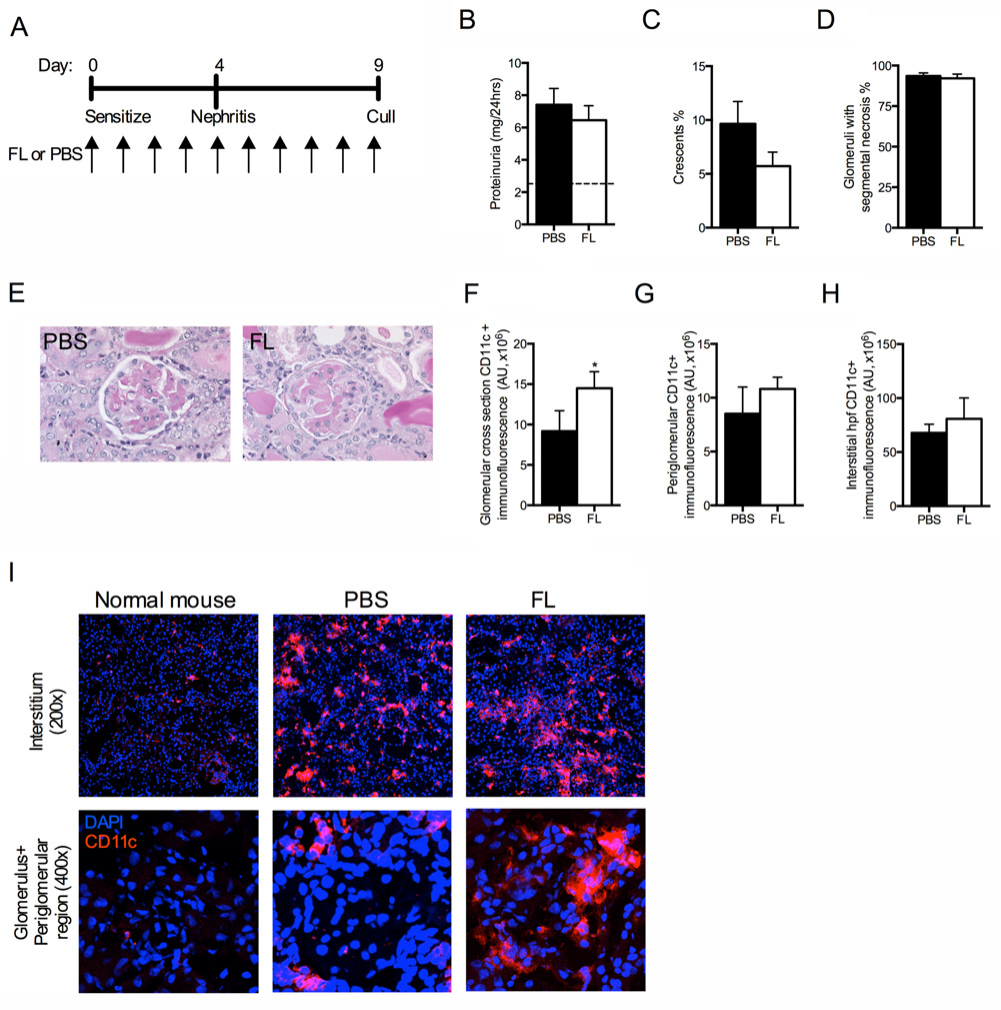
FL therapy throughout accelerated anti-GBM disease did not protect mice from nephritis. (A) Experimental design. (B) Proteinuria. (C) Percentage of glomerular crescents and (D) glomeruli with segmental necrosis. (E) Representative images of glomeruli from PBS and FL treated mice taken at high power (400x, PAS stain). (F–H) gcs, periglomerular and interstitial hpf CD11c+ immunofluorescence assessed by fluorescent microscopy of renal sections. (I) Representative images of CD11c+ immunofluorescent staining within the interstitial, glomerular and periglomerular regions. Black bars represent PBS treated mice. White bars represent FL treated mice. AU, arbitrary units; gcs, glomerular cross section; hpf, high-powered field. n = 7 per group. *P<0.05.

When FL treatment commenced on the day mice were sensitized to sheep globulin and continued through the course of disease (Fig 4A), more FL treated animals unexpectedly developed signs of renal failure, and met criteria for humane euthanasia, compared to PBS treated controls, occurring after sheep anti-mouse GBM globulin administration during nephritis (Fig 4B); the experiment was terminated early at day 10 of the model. A repeat experiment was performed, with animals being euthanized at day 9 (five days after anti-GBM administration; Fig 5A). Mice in both groups sustained significant functional renal injury (serum urea; PBS 121±16 vs FL 126±10 mmol/L, P = 0.78), with comparable proteinuria and histological damage, marked by widespread glomerular segmental necrosis (Fig 5B–5E). FL treated mice had enhanced interstitial neutrophil recruitment compared to controls (Table 2). Renal CD11c+ cells were increased within glomeruli of FL treated mice, with a trend to increase in periglomerular and interstitial regions (Fig 5F–5I).

**Table 2:** Intrarenal leukocytes in mice treated with PBS or FL throughout anti-GBM disease until day 9.

	Number of cells	
	PBS (n = 7)	FL (n = 7)
**Glomeruli (c/gcs)**		
Neutrophils	3.0 ± 0.3	2.4 ± 0.3
CD4+ T cells	0.2 ± 0.03	0.2 ± 0.03
CD68+ Macrophages	1.5 ± 0.2	1.7 ± 0.1
**Interstitium (c/hpf)**		
Neutrophils	11.4 ± 1.2	15.9 ± 1.3*
CD4+ T cells	9.8 ± 1.8	8.0 ± 1.2
CD68+ Macrophages	32.9 ± 2.5	34.7 ± 2.6

c/gcs, cells per glomerular cross section; c/hpf, cells per high power field.

*P = 0.02.

**Fig 6:**
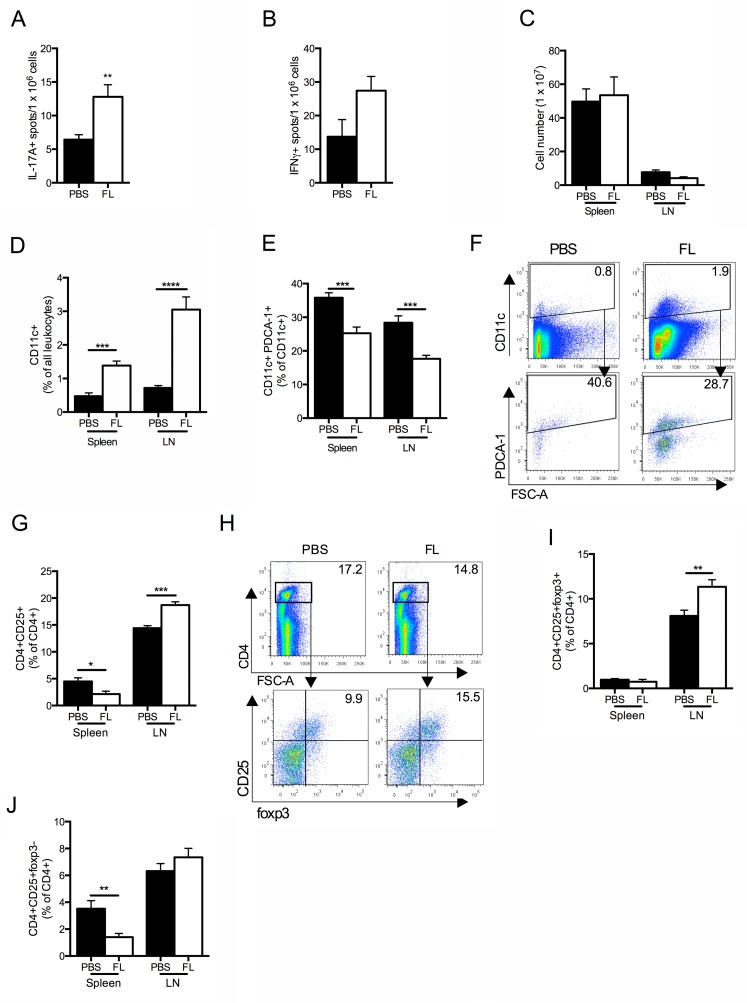
Cellular pro-inflammatory immune responses were heightened when FL was given during accelerated anti-GBM disease. (A) IL-17A+ spots and (B) IFNγ+ spots per 1 million stimulated splenocytes, measured by EliSpot. (C) Total spleen and LN cell number. (D, E) Proportion of CD11c+ cells and pDCs in the spleen and LN. (F) Representative FACS plots of splenocytes stained for CD11c and PDCA-1. (G) Proportion of activated T cells (CD4+CD25+/CD4+) in spleen and LN. (H) Representative FACS plots of pooled LN cells stained for CD4, CD25 and foxp3. (I, J) Proportions of Tregs (CD4+CD25+foxp3+/CD4+) and Teff (CD4+CD25+foxp3-/CD4+) in spleen and LN. Black bars represent PBS treated mice. White bars represent FL treated mice. n = 7 per group. *P<0.05, **P<0.01, ***P<0.001, ****P<0.0001.

Systemic inflammatory immune responses were heightened in the FL treated mice, with significantly increased IL-17A production, and a trend towards enhanced IFNγ production by splenocytes (Fig 6A and 6B). IL-6 concentrations were not different between groups (median with range, PBS 0 [0–12] vs FL 0 [0–14], P = 0.29). IL-12p70, TNF and IL-10 concentrations were undetectable in both groups. Within the spleen and LN, cell numbers were similar between groups, but FL treated mice had significantly increased proportions of CD11c+ cells and reduced proportions of pDCs (Fig 6C–6F). FL treated mice had elevated proportions of activated (CD4+CD25+) T cells and Tregs (CD4+CD25+foxp3+), with a similar proportion of effector T cells (CD4+CD25+foxp3-) within LN, but in the spleen FL treated mice had fewer activated T cells and Teff (Fig 6G–6J). Therefore, FL administration throughout experimental RPGN promoted maturation of DCs towards a conventional DC phenotype. It did not suppress nephritogenic immunity, but rather FL enhanced systemic immunity and T cell activation.

### FL administered before and during the induction of nephritogenic immunity

**Fig 7:**
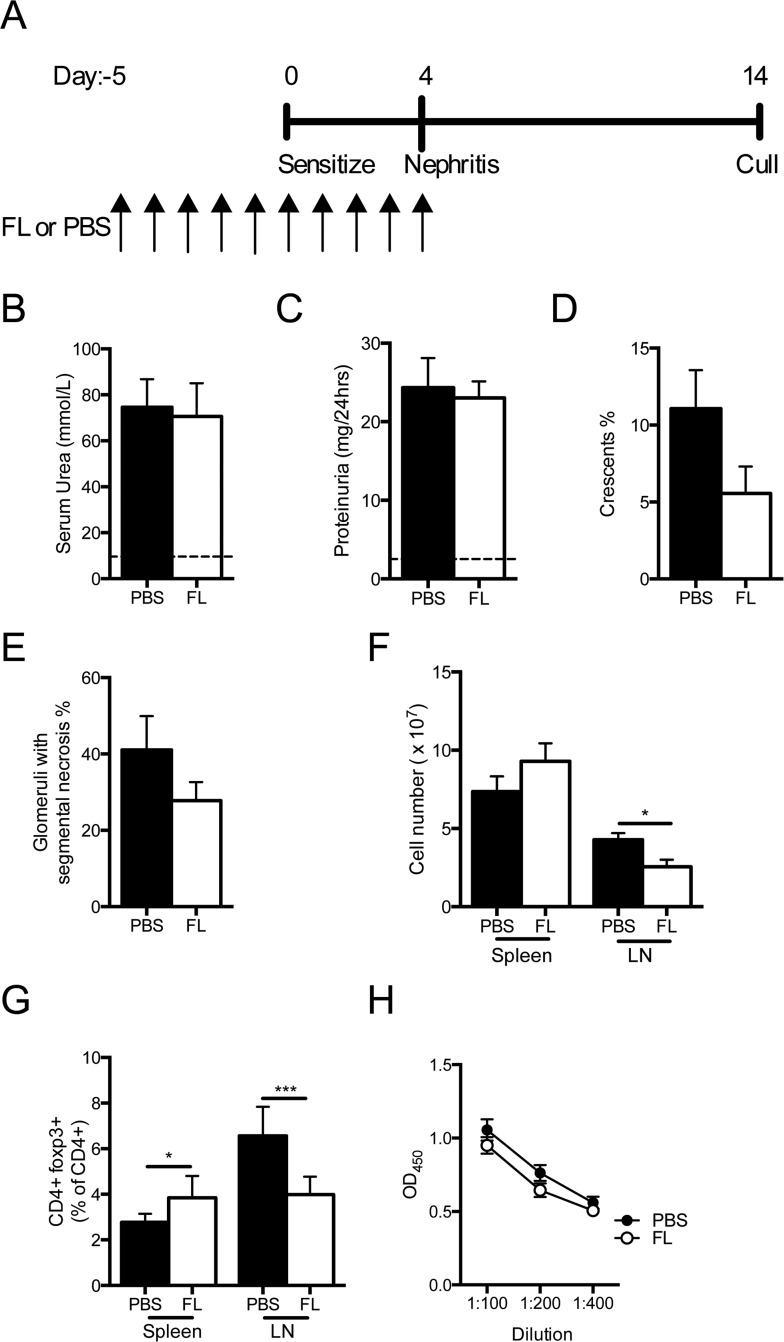
Effects of FL given during sensitization, but not during the nephritic phase of injury. (A) Experimental design. (B) Serum urea (dotted line represents measured level in non-nephritic WT mice, n = 4). (C) Proteinuria (dotted line represents measured level in non-nephritic WT mice, n = 4). (D, E) Percentage of glomerular crescents and segmental necrosis. (F) Total spleen and LN cell number. (G) Proportion of Tregs in the spleen and LN. (H) Serum mouse anti-sheep IgG antibody concentrations. Black bars represent PBS treated mice. White bars represent FL treated mice. OD, optical density. n = 7 for PBS group and 9 for FL group. *P<0.05, ***P<0.001.

This model of RPGN relies on planting sheep globulin, a foreign antigen, within the glomerulus of sensitized mice. It was possible that exogenous FL, administered at the time of anti-GBM globulin injection, enhanced the maturation and expansion of cDCs in sensitized mice rather than polarizing precursor DCs towards this phenotype. We sought to determine if FL could induce pDCs and enhance Treg populations in steady state conditions and suppress subsequent antigen-specific responses. Therefore, we administered FL before and during the sensitization period (i.e. when DCs were playing a key role in inducing immunity to sheep globulin) but discontinued it at the time nephritis was induced (Fig 7A).

Compared to controls, FL treated mice showed similar functional injury, with a trend towards fewer glomerular crescents and less segmental necrosis (Fig 7B–7E). There was no difference in splenocyte number between groups, but LN cell numbers were reduced in FL treated mice (Fig 7F). Splenic regulatory T cell populations of FL treated animals were enhanced, but fewer Tregs were present in the LN of FL treated mice (Fig 7G). There were no significant differences in the proportions of CD11c+ cells and pDCs between groups (CD11c+ spleen PBS 1.3±0.3 vs FL 0.8±0.1%, P = 0.11; CD11c+ LN PBS 1.9±0.1 vs FL 1.8±0.3, P = 0.8 and pDCs spleen PBS 1.4±0.6 vs FL 1.3±0.7%, P = 0.91; pDCs LN PBS 4.5 ±1.2 vs FL 5.3±1.2%, P = 0.67). There was no difference in IFNγ, IL-17A, IL-4 or IL-10 concentrations in stimulated splenocyte supernatant (Table 3); IL-6, IL-12p70 and TNF concentrations were undetectable. Serum antigen specific IgG antibodies between groups were not different (Fig 7H). Therefore, FL administration before and during the sensitization phase did not result in enhanced injury as seen when FL was given throughout the nephritis phase, but did not protect mice from effector T cell mediated renal injury.

**Table 3:** Cytokine concentrations in stimulated splenocyte supernatant for mice receiving PBS or FL before and during sensitization to the nephritogenic antigen in the anti-GBM disease model.

	Concentration (pg/mL)		
Cytokine	PBS (n = 7)	FL (n = 9)	P
IL-17A	173 ± 49	200 ± 77	0.78
IFNγ	332 ± 45	447 ± 79	0.3
IL-4	1100 ± 213	711 ± 227	0.24
IL-10	22 (0–218)	0 (0–74)	0.57

Data presented as mean±SEM, except for IL-10, presented as median (range) as data is non-parametric. IL-6, IL-12p70 and TNF were not detected.

## Discussion

We have shown that FL administered under homeostatic conditions expands Treg and DC populations. DC expansion was not restricted to pDCs, but both cDC and pDC populations were increased. Despite inducing Tregs, FL given prior to autologous phase accelerated anti-GBM GN did not result in significant protection from renal injury, although we found evidence that some elements of systemic immunity were attenuated, with reduced IL-17A production from splenocytes. When FL was given throughout the nephritis model, FL treated mice exhibited excess mortality. This was likely, but not certain, to be due to renal disease. We repeated this model, euthanizing mice earlier to assess renal injury and systemic immune responses prior to mice becoming too unwell. We found similar, but severe, glomerular injury in both groups. However, glomerular CD11c+ DCs and neutrophil recruitment to the renal interstitium was increased in FL treated mice compared to controls, indicating greater pro-inflammatory local immune responses had developed in the FL group. Systemic immune responses were also increased in the FL group, with enhanced Th17 cellular immunity and increased cDC populations in secondary lymphoid organs. Therefore, FL administration throughout RPGN polarized naïve T cells towards an effector rather than a regulatory phenotype.

Despite the persistence of a higher proportion of pDCs after FL had been given for 10 days, mice subsequently sensitized to sheep globulin had enhanced dermal DTH 4 days later. Therefore, despite enhanced Treg populations and pDCs at the time of sensitization, the induction of immunity to sheep globulin enhanced effector T cell responses, indicating effective antigen presentation by the DC populations. When FL was given before and during sensitization, but not during nephritis, renal injury or systemic immune responses were not altered. These findings suggest that when FL was given in steady-state conditions, expanded DC populations and Treg induction were capable of modulating effector T cell responses and cytokine release.

There are variable reports of the effectiveness of FL therapy in experimental autoimmune disease. In the NOD mouse type I diabetes model, pancreatic beta cell destruction was prevented by administering FL prior to the development of pancreatic injury in young, but not in older mice, despite increasing DC and Treg populations in spleen and pancreatic LN [32, 33]. The onset of diabetes was accelerated by FL therapy given to NOD mice with detectable auto-reactive T cells with increased pro-inflammatory cytokine expression [33]. FL attenuated inflammatory bowel disease in the TNFΔARE mouse model, by enhancing Tregs [34].

In models of T cell dependent inflammation or asthma/airways disease induced by foreign antigens, FL therapy has also provided mixed results. Experimental methylated bovine serum albumin induced arthritis was not significantly attenuated by FL treatment [35], whereas treatment with sumatinib (a tyrosine kinase receptor inhibitor directed against the FLT3 receptor) protected mice from joint damage [35, 36]. FL therapy did protect mice from allergic airways inflammation, with evidence for either a Th2 to Th1 shift in immunity [37, 38] or enhanced Treg function and recruitment [39, 40]. Similarly, experience with FL in models of transplantation provides conflicting results. Allogeneic T cell responses were reduced and mouse survival increased when bone marrow recipients were pre-treated with FL in a model of experimental graft vs host disease [15], and when FL treated renal DCs were transferred into mice receiving allogeneic cardiac transplants [13]. However, FL administration to donors prior to liver transplantation expanded myeloid and lymphoid DCs in the grafts, worsened graft survival, with evidence of heightened Th1 immune responses and less apoptosis of alloreactive T cells [41].

*In vitro* and *in vivo* studies suggest DC maturity directs their suppressive or inflammatory capacity. In steady state, DCs from mice receiving FL had an immature phenotype and were poor stimulators of T cell proliferation in co-culture *ex vivo*, but they could induce expansion of regulatory cells [13, 15, 42, 43], without requiring MHCII expression [15]. However, lipopolysaccharide (LPS)-matured FL-induced DCs potently induced naïve T cell proliferation without Treg expansion [13]. Bone marrow derived DCs cultured with FL plus IFNα or LPS had increased surface expression of activation markers and enhanced T cell proliferation compared to bone marrow DCs cultured with FL alone. Therefore, in an inflammatory milieu, FL treated DCs mature and take on a more conventional phenotype [42]. Similarly, compared to FL treated mice receiving peptide immunisation alone, FL treated mice receiving immunisation with peptide and adjuvant (FCA) had enhanced T cell activation and proliferation, and more DCs with a mature phenotype [44]. In the presence of toll like receptor 9 agonists or inactivated influenza virus, pDCs transformed into cells exhibiting a cDC phenotype, with increased IFNα production and the capacity to stimulate T cells in vitro [45]. Transfer of immature FL treated DCs into mice prior to experimental autoimmune encephalitis (EAE) induction (with myelin oligodendrocyte glycoprotein/FCA) resulted in less severe disease than mice pre-treated with TNF and LPS matured FL DCs [46]. When FL therapy was initiated five days prior to EAE induction and continued for a further five days, FL treated mice developed increased disease severity compared to controls [47], whereas treatment of mice with established EAE with a FL inhibitor (CEP-701, which induced apoptosis of mature DCs) resulted in improvement in disease progression [48]. These studies suggest that although FL therapy promotes immature DCs and Tregs in steady state, these DCs can become polarised towards a conventional phenotype in the presence of pro-inflammatory cytokines and proteins. This may explain some of the conflicting reports about the immunomodulatory effects of FL, and why FL was not protective when given during our experimental crescentic GN model, which relies on an adaptive immune response to sheep globulin planted along the GBM in sensitized mice [46].

The current studies have shown that FL administered throughout a murine model of RPGN heightened inflammatory effector T cell immune responses, resulting in reduced animal survival. The pitfalls of FL therapy demonstrated in this work highlight the challenges inherent in manipulating cellular immunity to promote Tregs in immune renal disease.
